# Green Rubber Technology: The Potential of Ophthalmic Lens Waste as a Filler in Styrene–Butadiene Rubber-Based Composites

**DOI:** 10.3390/ma18081842

**Published:** 2025-04-17

**Authors:** Carlos Toshiyuki Hiranobe, Elmer Mateus Gennaro, Guilherme Henrique Barros de Souza, Dener da Silva Souza, Samara Araújo Kawall, Márcia Ferreira Hiranobe, Leandra Oliveira Salmazo, Miguel Angel Rodríguez Pérez, Alberto Lopez Gil, Eduardo Soares Nascimento, Erivaldo Antônio da Silva, Renivaldo José dos Santos

**Affiliations:** 1 Department of Engineering, School of Engineering and Sciences, São Paulo State University (UNESP), Rosana Campus, Avenida dos Barrageiros, Rosana 19274-000, SP, Brazil; carlos.hiranobe@unesp.br (C.T.H.); dener.souza@unesp.br (D.d.S.S.); s.araujo@unesp.br (S.A.K.); 2 Department of Aeronautical Engineering, Engineering School, São Paulo State University (UNESP), Avenida Professora Isette Corrêa Fontão, São João da Boa Vista 13876-750, SP, Brazil; elmer.gennaro@unesp.br; 3 Department of Cartographic and Surveying Engineering, School of Science and Technology, São Paulo State University (UNESP), Presidente Prudente Campus, Rua Roberto Simonsen, Presidente Prudente 19060-900, SP, Brazil; guilherme.barros@unesp.br (G.H.B.d.S.); e.nascimento@unesp.br (E.S.N.); erivaldo.silva@unesp.br (E.A.d.S.); 4 Department of Tourism and Territory Development, School of Engineering and Sciences, São Paulo State University (UNESP), Rosana Campus, Avenida dos Barrageiros, Rosana 19274-000, SP, Brazil; marcia.hiranobe@unesp.br; 5 Cellular Materials Laboratory (CellMat), Condensed Matter Physics Department, University of Valladolid, Paseo de Belén 7, 47011 Valladolid, Spain; leandra.salmazo@fmc.uva.es (L.O.S.); marrod@fmc.uva.es (M.A.R.P.); a.lopez@cellmattechnologies.com (A.L.G.)

**Keywords:** styrene–butadiene rubber, eco-efficient composites, Lorenz–Parks, polymethyl methacrylate, ophthalmic lens waste

## Abstract

The article examines the possibility of using ophthalmic lens waste (OLW) as a filler in styrene–butadiene rubber (SBR) composites in varying proportions. It analyzes the impact of OLW on the composites’ rheological, structural, morphological, mechanical, and thermal properties. Results show that OLW addition does not significantly alter vulcanization time, leading to thermal savings during processing. The crosslink densities, determined by the swelling method in an organic solvent and the mechanical behavior of the elastomers, increased with the incorporation of OLW, suggesting a filler/polymeric matrix interaction. The Lorenz–Parks model confirmed matrix–filler interaction, although it was insufficient to substantially improve mechanical reinforcement, with OLW mainly acting as a filler. Thermogravimetric tests revealed good thermal stability, but dynamic mechanical analysis indicated reduced damping properties. Spectroscopic analysis indicated the lack of molecular bonding between the polymer and the OLW filler. The study suggests that the optimal OLW content is between 10 and 20 phr, enabling the production of a new composite. Overall, incorporating OLW into vulcanized SBR composites offers a sustainable and cost-effective approach to reusing industrial waste in polymer production, providing an environmentally friendly alternative for the polymer industry.

## 1. Introduction

The World Report on Vision by the World Health Organization (WHO), published in 2019, reveals alarming data: it is estimated that at least 2.2 billion individuals globally experience some degree of visual impairment or loss of sight. Among these, at least 1 billion have a problem that could have been prevented or has not yet been treated [1]. Myopia, commonly known as “short-sightedness”, has shown a significant increase, particularly among the younger population. This growing prevalence is directly related to the excessive time children and young people spend in front of digital devices. Conversely, the aging population drives the demand for single-vision lenses to correct presbyopia, an age-related eye condition. Factors such as lifestyle and fashion also influence the choice and consumption of ophthalmic lenses, highlighting the need for personalized solutions that meet the specific needs of everyone [2].

Keeping pace with the accelerated demand, the ophthalmic lens market is expected to show a compound annual growth rate (CAGR) of 4.85% from 2022 to 2027. In monetary terms, this translates to a USD 14.3 billion increase in the lens market size [3]. In addressing this growth, it is crucial to consider the environmental implications of increased production. The rising demand for lenses will lead to a proportional increase in generated waste, which can cause negative environmental impacts if not properly managed. Materials used in ophthalmic lens production, such as glass, plastic, and acrylic, have a long decomposition time and can pollute landfills and oceans [4]. Although precise global data on the amount of ophthalmic lens waste (OLW) generated annually is not available, it is evident that the industry recognizes its environmental impact and has adopted various strategies to mitigate it.

To minimize these impacts, it is essential to adopt measures that promote the reduction, reuse, and recycling of waste generated from ophthalmic lenses. Studies conducted by Perez et al. [5] highlight that plastics are the primary material used in the manufacturing of lenses and eyewear in the ophthalmic industry. However, during the production of prescription lenses, up to 90% of the thermoset material is wasted, making it unsuitable for recycling. To improve the overall energy balance and minimize environmental impact, several strategies have been proposed. These include reducing the total manufacturing time, enhancing the efficiency of scratch-resistant treatments, improving vacuum coating systems, and utilizing data generated throughout the production process. Furthermore, the recycling of waste generated from processing materials emerges as a viable alternative. It is argued that the implementation of these measures can significantly contribute to reducing energy consumption, carbon footprint, waste generation, and operational costs in the manufacturing of ophthalmic lenses and eyewear.

The approach proposed by Encarnação et al. [6] involves integrating solid waste materials from ophthalmic lenses into polymer and cement matrices, alongside using effluent for cultivating microalgae. This pioneering approach concentrates on biomass production and the economic enhancement of waste. By implementing a circular economy framework, the objective is to transform ecological contaminants found in the effluent into valuable organic products. Batista et al. [7] investigated the feasibility of creating composites using bamboo powder and OLW combined with polyester resin. These materials are often discarded in large quantities and have the potential to be reused in an environmentally sustainable manner. Tests were conducted to determine the optimal proportions of each material in the composite composition, aiming to ensure good processability and suitable mechanical properties. The results indicated that the produced composites exhibited a reduction in mechanical resistance relative to the resin matrix but demonstrated a substantial enhancement in impact resistance. For practical implementation, tables and benches were fabricated using the most cost-effective and environmentally sustainable composite composed of 40% OLW.

Considering the above, it is evident that studies are directed toward environmental awareness and reducing water consumption, electricity, and the volume of waste produced. Nevertheless, limited focus has been given to the management of solid waste produced during the fabrication of ophthalmic lenses. To address this gap, this study aims to explore the potential of OLW to enhance the properties of vulcanized rubber composite, especially styrene–butadiene rubber (SBR). The effects of incorporating OLW on rheometric, structural, and morphological properties were analyzed. SBR/OLW composites were subjected to various analytical conditions, evaluating mechanical and thermal responses, as well as the reinforcement degree between the filler and elastomeric matrix.

## 2. Procedures and Techniques

### 2.1. Materials

The manufactured styrene–butadiene elastomer (SBR 1502), featuring a Mooney viscosity above 49.0 (tested at 100 °C) and a bonded styrene content of 23.5%, was procured from DLP Rubber and Artifacts Industry and Trade LTDA (Poloni, SP, Brazil). The ophthalmic lens waste (OLW) was supplied by Perego Lens Industry and Trade LTDA (Araçatuba, SP, Brazil). The specified waste material was dried at 100 °C and sieved to retain particles larger than 200 mesh. All essential curing additives—including zinc oxide (Vetec, Duque de Caxias, RJ, Brazil), stearic acid (Synth, Diadema, SP, Brazil), polyethylene glycol (PEG 4000; Sigma-Aldrich, St. Louis, MO, USA), Chartwell® bonding agent (Chartwell International, Ashland, OH, USA), extender oil (Quimisul, São Paulo, SP, Brazil), sulfur (Quimidrol, Joinville, SC, Brazil), and accelerators such as benzothiazole disulfide (MBTS) and tetramethyl thiuram sulfide (TMTD) (Basile Química, São Paulo, SP, Brazil)—were acquired from certified suppliers known for high-purity standards. Table 1 outlines the formulation used to produce the SBR/OLW blends.

#### Method for Producing Composite Materials

The mixtures were processed in a two-roll mill blender in accordance with ASTM D3182-21a standards [8] using a friction ratio of 1:1.25. The proportions commonly used in vulcanized rubber composites were 0, 10, 20, 30, 40, and 50 phr. Above this value, the mixture becomes difficult to process and is brittle, compromising the properties of the composite. Styrene–butadiene rubber, curing activators (zinc oxide and stearic acid), and softening agents (polyethylene glycol 4000 and naphthenic processing oil), along with ophthalmic lens waste as a filler, were mixed in the two-roll mill. Once the blend was uniformly mixed for 20 min, it was left to cure for 24 h under ambient conditions (23 °C). Next, the compound was returned to the mixing chamber for incorporation of the curing agent (sulfur) and vulcanization promoters (MBTS and TMTD). After thorough blending for 15 min, the material rested for an additional 2 h at ambient temperature. Following these procedures, the resulting composite was subjected to rheological analysis before being molded in a heated press to fabricate testing samples.

### 2.2. Techniques Employed

The techniques employed are listed below. All tests were conducted at least in triplicate to ensure the reproducibility of the results.

#### 2.2.1. Determination of Rheological Parameters

The rheological characteristics were analyzed using an oscillatory disk rheometer manufactured by Team Equipamentos do Brasil. Tests followed ASTM D2084-17 guidelines [9], which involved subjecting the composite to a 1° oscillation arc and a constant temperature of 160 °C. After obtaining rheological parameters, the SBR/OLW composites underwent the thermoforming process. This procedure was conducted using a Mastermac press, Vulcan 400/20-1 model manufactured in São Paulo, Brazil, with a maximum pressure of 210 kgf·cm^−2^, along with a 1010/1020 steel mold (150 × 150 × 2 mm).

#### 2.2.2. Evaluation of Filler Dispersion Homogeneity Within the Polymeric Matrix

The homogeneity in particulate distribution throughout the elastomeric network serves as an indicator of dispersion quality. Optimal uniformity reflects consistent morphological characteristics of the dispersed filler, which may improve multiple performance characteristics of the composite material, such as wear resistance, surface durability, and crack propagation resistance. The quantitative analysis of filler dispersion characteristics for ophthalmic lens waste within elastomeric blends can be determined using Equation (1) [10]:

(1) $$L=\frac{M_{Lf}}{M_{Lg}}-\frac{M_{Hf}}{M_{Hg}}$$

where *L* indicates the dispersion level of particulate filler within the polymeric network; *M_L_* corresponds to the lowest recorded torque value; *M_H_* represents the peak torque measurement; *f* and *g* signify the filled compound and unfilled base polymer, respectively.

#### 2.2.3. Electron Microscopy Analysis

The fracture surface characteristics of the composite materials were examined with a Carl Zeiss EVO LS15 electron microscope operated at 20 kV, produced in Oberkochen, Germany. Specimens were deposited with a conductive gold film employing a Quorum Q 150R ES deposition system (Laughton, UK).

#### 2.2.4. Measurement of Composite Material Density

The evaluation of composite material density was conducted according to ASTM D297-21 standards [11], employing ethanol having a specific gravity of 0.79 g·cm^−3^. The density was computed using Equation (2):

(2) $$\rho =\frac{\rho_{L}*\;m_{A}}{m_{A}-m_{B}}$$

where *ρ* denotes the specimen density (g cm^−3^); *ρ_L_* corresponds to the ethanol density at test conditions (g cm^−3^); *m_A_* indicates the specimen mass (g); and *m_B_* refers to the specimen mass when immersed (g).

#### 2.2.5. Evaluation of Network Connectivity in Composite Materials

The polymer network density of the specimens was assessed through solvent swelling methodology. Test samples weighing approximately 0.25 ± 0.05 g were submerged in toluene for five days. Following immersion, the samples were extracted, surface-dried to eliminate residual solvent, and reweighed. Subsequent oven drying at 80 °C for 24 h preceded final mass measurements. The collected data, including initial mass, solvent–swollen mass, and post-drying mass, enabled computation of the elastomer volume fraction in the swollen matrix. Network density quantification employed the Flory–Rehner equation (Equation (3)) [12], with toluene’s molar volume (*V*_0_) and the Flory–Huggins parameter (χ) for the styrene–butadiene–toluene system established as 106.3 cm^3^ mol^−1^ and 0.393, respectively.

(3) $$\nu =\frac{-(\ln\left(1-V_{B}\right)+V_{B}+\chi \left(V_{B}{)}^{2}\right)}{\left(\rho_{B}\right)\left(V_{0}\right)\left(V_{B}^{\frac{1}{3}}-\frac{V_{B}}{2}\right)}$$

In this equation, *ν* represents the network chain concentration (mol cm^−3^); *ρ_B_* denotes the elastomer’s specific gravity (g cm^−3^); and *V_B_* corresponds to the polymeric volume fraction in the swollen state, calculated from the mass variation during swelling.

The network densities were additionally calculated through the Mooney–Rivlin approach [13] utilizing uniaxial tensile data. To construct the linear elastic response curve and determine the structural constants, the semi-empirical Equation (4) was applied [14]:

(4) $$\sigma =\frac{F}{2A_{0}\left(\lambda -\lambda^{-2}\right)}=C_{1}+\frac{1}{\lambda }C_{2}$$

In this equation, *F* represents the applied force on the cured compound; *A_0_* denotes the initial specimen area (mm^2^); *λ* indicates the deformation ratio (1 + ε), with ε being the mechanical strain; *C*_1_ and *C*_2_ are polymer-specific coefficients, where *C*_1_ reflects the network junction contribution, and *C*_2_ corresponds to the Mooney–Rivlin elasticity parameter, representing constrained chain interactions.

The coefficient *C*_1_ is used in determining the crosslink densities and can be employed in formulating Equation (5) [15]:

(5) $$\eta =\frac{C_{1}}{\mathrm{RT}}$$

In this relationship, η denotes the network chain concentration (mol cm^−3^); R represents the ideal gas constant; and T indicates the thermodynamic temperature (K).

#### 2.2.6. X-Ray Spectroscopic Examination of Composite Materials

The elemental constitution of the specimens was characterized by X-ray fluorescence spectroscopy, employing a Malvern Panalytical spectroscopic instrument, Axios line, model PW 4400/40, produced in the Almelo, Overijssel, The Netherlands. Data processing was performed using SUPERQ 5.1B analytical software within the Omni platform. This technique yields data regarding the material’s chemical constituents in quantitative and qualitative formats, detecting present elements and their concentrations.

#### 2.2.7. Infrared Spectroscopic Analysis

Fourier-transform infrared analysis was conducted with a Bruker FTIR system, model Invenio, produced in Ettlingen, Baden-Württemberg, Germany, utilizing attenuated total reflection (ATR) methodology, scanning the spectral region from 4000 to 400 cm^−1^, with 4 cm^−1^ resolution and 32 accumulated scans.

#### 2.2.8. Evaluation of Mechanical Properties in Composites

Tension and tearing resistance measurements were carried out on a Brazilian-made Biopdi universal tester at 500 mm min^−1^, equipped with 5 kN load measurement capability and a built-in strain gauge. For the tensile property determination, five replicates of ASTM Type A specimens (narrow-waisted samples) were tested per ASTM D412-16 [16]. Tear resistance evaluation employed five replicates of Type C specimens (trouser-cut design) following ASTM D624-00 [17].

#### 2.2.9. Assessment of Composite Material Hardness

The measurement of material surface rigidity for the prepared specimens was conducted according to ASTM D2240-15 [18], employing an analog hardness tester (Digimess, São Paulo, SP, Brazil). Measurements were taken using the Shore A hardness scale (0–100 range) with 1-unit precision.

#### 2.2.10. Friction Resistance Test of the Composites

The determination of abrasion loss was conducted using Equation (6), as per ASTM D5963-04 [19]. For this purpose, the equipment from MaqTest, manufactured in Brazil, was utilized, with an abrasion path of 40 m and a pressure of 5 N applied to the specimen in contact with the cylinder.

(6) $$PA=\frac{\Delta m*S_{0}}{\rho \;S}$$

In this equation, *PA* denotes the volumetric wear caused by frictional wear (mm^3^/40 m); Δ*m* corresponds to the composite’s mass reduction (mg); *S*_0_ represents the reference abrasion coefficient for sandpaper against control rubber (200 ± 20 mg); *S* indicates the actual abrasion coefficient of the sandpaper on control rubber (mg); and *ρ* signifies the composite’s specific mass (mg.mm^−3^).

#### 2.2.11. Analysis of the Interactions Between Styrene–Butadiene Rubber and Ophthalmic Lens Waste Using the Lorenz–Parks Equation

The interfacial compatibility between ophthalmic residues and the styrene–butadiene elastomer was evaluated following the protocol established by Lorenz–Park [20], with parameters derived from swelling experiments through Equation (7) [21]:

(7) $$\frac{Q_{f}}{Q_{g}}=ae^{-z}+b$$

In this relationship, *Q* denotes the toluene uptake per unit mass of elastomer; subscripts *f* and *g* identify the filled composite and unfilled rubber matrix, respectively; *z* represents the filler-to-rubber mass ratio; and “*a*” and “*b*” are empirical coefficients. The *Q* value is determined through Equation (8):

(8) $$Q=\frac{w_{s}-w_{d}}{w_{r}\;x\;100/w_{F}}$$

In this equation, *w_s_* indicates the equilibrium mass of the swollen specimen; *w_d_* represents the mass of the dehydrated sample; *w_r_* corresponds to the elastomer content in the dry composite; *w_F_* signifies the total mass of the composite formulation.

#### 2.2.12. Thermogravimetric Evaluation of Polymer Composites

Thermal stability assessments were performed using a Netzsch thermal analyzer, model TG 209, of Selb, Bavaria, German origin. The evaluation spanned a thermal gradient between ambient temperature (25 °C) and 900 °C, employing a constant temperature ramp of 10 °C per minute in an inert nitrogen environment maintained at 15 mL/min flow rate. Specimen mass for analysis averaged approximately 10 mg, following ASTM protocol D6370-99 [22].

#### 2.2.13. Evaluation of Viscoelastic Properties for the Composites

The tests for dynamic mechanical analysis (DMA) were carried out using a Netzsch DMTA 242C device, produced in Selb, Bavaria, Germany, operating in tensile mode at a constant oscillation frequency of 10 hertz, with a thermal increment of 10 °C·min^−1^ and a controlled temperature span ranging from −100 °C up to 150 °C, on samples measuring approximately 10 × 5 × 0.25 mm.

## 3. Results and Discussion

### 3.1. Rheological Property Analysis

Table 2 displays the rheological characteristics. Higher filler loading resulted in a gradual elevation of minimum torque readings, reflecting enhanced compound viscosity. During thermal processing, network formation develops, causing maximum torque augmentation. Torque fluctuations correlate with both crosslinking development and filler incorporation. Notwithstanding measurement deviations, torque changes showed minimal variation regardless of filler content. Comparable trends were observed for the induction period and t_90_ relative to the control formulation (0 phr). Notably, the induction period represents the thermal exposure interval prior to complete crosslinking, whereas t_90_ indicates the time required for 90% bond formation between polymer chains. These findings suggest that filler incorporation neither modifies curing kinetics nor affects processing characteristics.

### 3.2. Assessment of Filler Distribution Homogeneity in Recycled Lens Waste-Reinforced SBR Compounds

**Figure 1:**
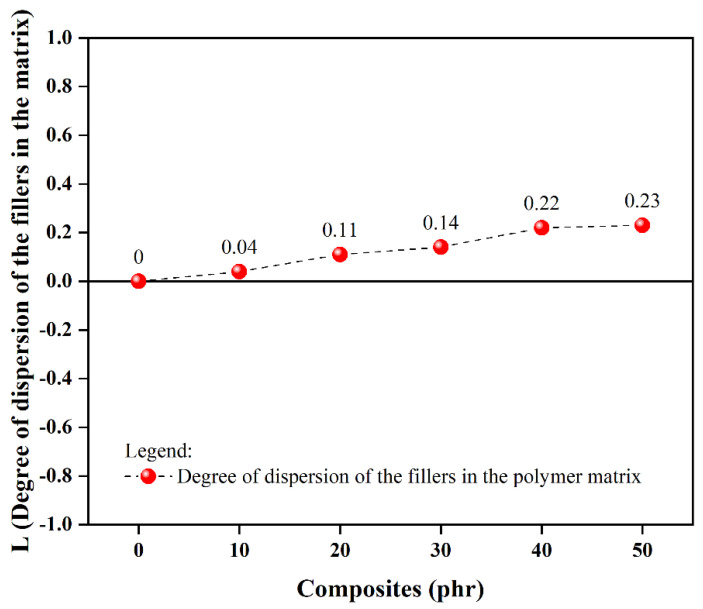
Degree of dispersion of OLW fillers in the SBR matrix.

Figure 1 illustrates the relative dispersion characteristics of ophthalmic waste particles within the styrene–butadiene elastomer network, using the unfilled compound (0 phr) as the baseline. Values approaching the reference indicate superior filler distribution. Results demonstrate acceptable dispersion stability up to 30 phr waste incorporation. However, beyond 40 phr loading, particle clustering becomes evident, negatively impacting material performance. The analysis further reveals that decreased system viscosity correlates with both reduced minimum torque and improved particulate distribution, promoting interfacial adhesion between the recycled filler and rubber matrix. This enhanced compatibility manifests as supplementary physical network junctions that augment the total crosslink density.

### 3.3. Electron Microscopy Analysis

**Figure 2:**
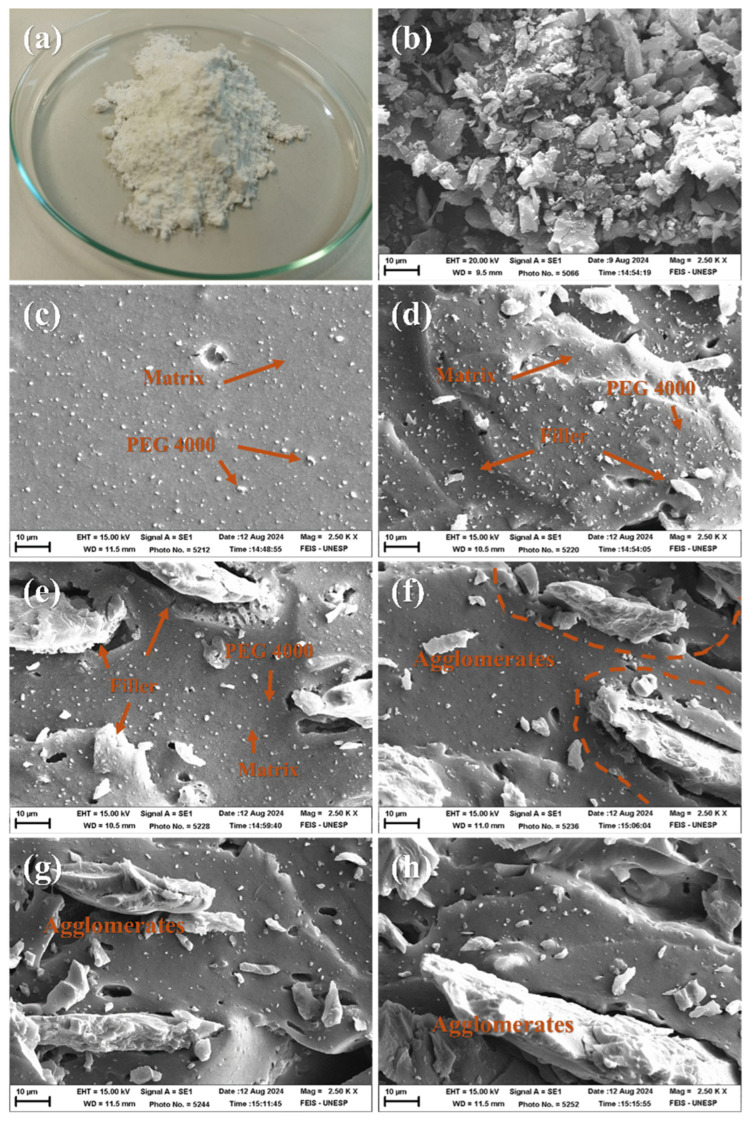
(**a**) Photographic image of ophthalmic lens waste; SEM images at a scale of 10 µm and magnification of 2.5 k× (**b**) of the ophthalmic lens waste; fracture regions of the tensile test specimens of the composites containing (**c**) 0 phr, (**d**) 10 phr, (**e**) 20 phr, (**f**) 30 phr, (**g**) 40 phr, and (**h**) 50 phr of lens waste.

Figure 2 presents photographic images of OLW and scanning electron micrographs of the lens waste and their composites obtained from the fracture regions of the tensile test specimens. In Figure 2a, a photographic image of the OLW obtained from the grinding and polishing process of the lenses after drying in an oven at 100 °C and sieving through a 200-mesh screen is shown. Figure 2b–h show scanning electron microscopy (SEM) images at a scale of 10 µm and a magnification of 2.5 k×. Figure 2b displays the micrograph of the OLW, where particles of various sizes and shapes can be observed, resembling terrestrial rocks with smooth edges. Figure 2c shows the SEM image of the unfilled composite at the fracture region of the specimen, where a smooth and homogeneous surface is observed, with small granules dispersed in the SBR rubber matrix. These granules can be attributed to the plasticizing agent polyethylene glycol 4000, which functions to improve the flexibility and elasticity of the composite. Figure 2d shows the SEM image of the SBR composite with 10 phr of OLW, where a homogeneous surface is observed, albeit with elevations and depressions, as well as the presence of dispersed fillers in the polymer matrix. Figure 2e–h refer to the composites with 20, 30, 40, and 50 phr of OLW, respectively. In these figures, it is possible to observe that the fillers are merely adhered to the matrix without anchoring points that prevent their extraction. Furthermore, starting with the composite containing 20 phr of OLW, the excess fillers tend to form stress points that weaken the bonds between the polymer chains, causing them to break more easily.

### 3.4. Evaluation of X-Ray Fluorescence of Ophthalmic Lens Waste

The results regarding the chemical composition of OLW were obtained through X-ray fluorescence tests, as detailed in Table 3. It is notable that the sample consists predominantly of carbon, along with the presence of some alkali metals that have the potential to enhance accelerator action, such as calcium and potassium, as well as zinc, which act as activators in the vulcanization process. Furthermore, sulfur is present, facilitating the establishment of three-dimensional networks among the elastomer’s polymer chains. Notably, silica derived from silicon carbide or silicon dioxide decomposition during grinding is also detected. This component can enhance structural reinforcement within the polymeric network, consequently improving the mechanical performance of the vulcanized material [23].

### 3.5. Characterization of Mass Density, Indentation Resistance, and Surface Degradation in Hybrid Materials

**Figure 3:**
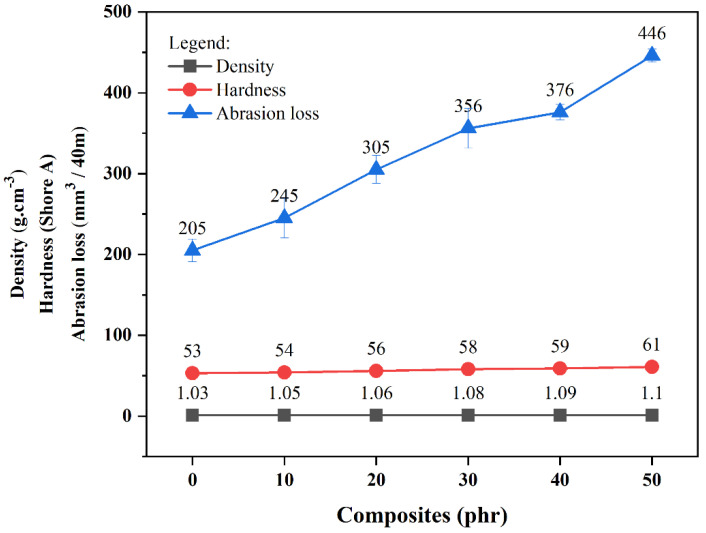
Density, hardness, and abrasion loss curves of the composites.

The data presented in Figure 3 demonstrate the trends and experimental findings from density determinations, Shore A hardness tests, and abrasion resistance measurements of the engineered composites. Observations reveal a progressive density enhancement with increasing filler concentration, spanning 1.0–1.1 g·cm^−3^. Indentation resistance likewise improved with particulate inclusion, as the additives restrict elastomer chain mobility, causing structural reinforcement. Oppositely, surface wear showed marked elevation at greater filler levels, attributable to stress concentration zones that accelerate particle liberation during sliding contact.

### 3.6. Evaluation of the Tensile Strength of the Composites

**Figure 4:**
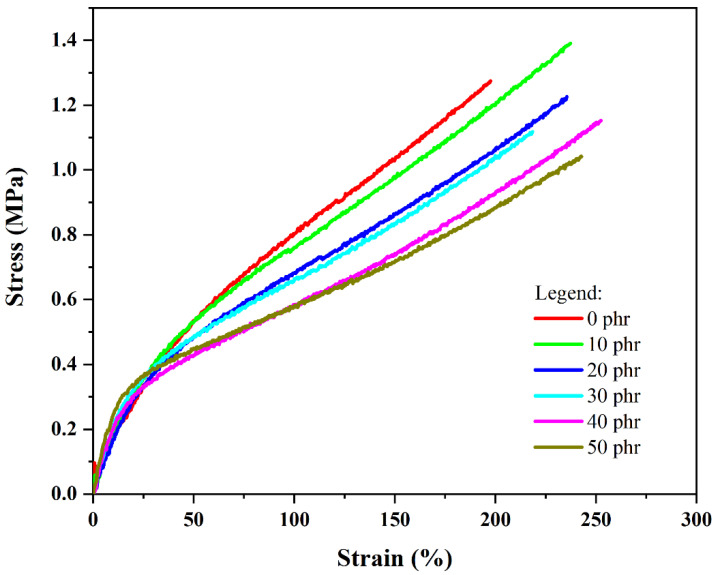
Tensile strength curves of SBR/OLW composites.

Figure 4 presents the stress–strain profiles, while Table 4 summarizes the experimental data from uniaxial tensile testing of styrene–butadiene/optical waste formulations at failure point.

It is observed that the 100% and 200% modulus of elasticity tend to decrease with the addition of fillers, resulting in a stiffer composite, as evidenced by the values presented in Table 4 and the profile of the curves shown in Figure 4. Concerning the ultimate tensile properties, filler incorporation appears to have minimal impact on the reinforced materials relative to the control sample, indicating the additives primarily function as space-filling constituents rather than strengthening agents.

### 3.7. Evaluation of the Tear Resistance of Composites

**Figure 5:**
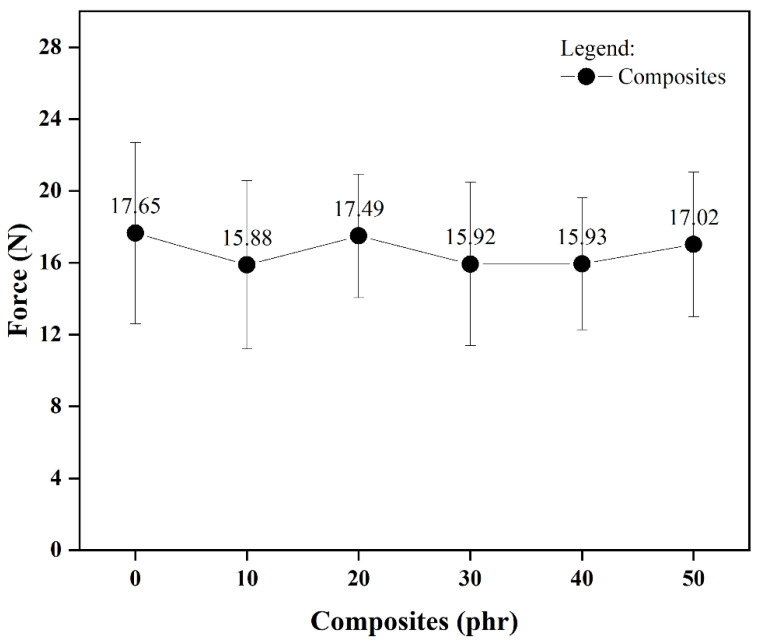
Tear resistance curves of SBR/OLW composites.

The experimental data from fracture propagation resistance evaluations appear in Figure 5. Consistent with the uniaxial tension results, observations indicate that higher filler loading shows negligible effect on tearing resistance relative to the control formulation. This suggests that the fillers do not act as barriers that impede or reduce tear propagation.

### 3.8. Network Connectivity Assessment via Solvent Swelling (Flory–Rehner Method)

The establishment of molecular networks serves as a fundamental factor in rubber characterization. Quantifying network densities offers valuable information that enables optimization of composite performance characteristics under mechanical and thermal stresses [24]. Determination of network connectivity through solvent immersion until equilibrium, applying the Flory–Rehner theoretical framework, produces reliable data [25]. The calculated network densities using this methodology are presented in Table 5. Systematic enhancement of network connectivity within the material system occurs following additive incorporation, demonstrating successful filler–matrix compatibility. However, the methodology cannot distinguish chemical linkages between elastomer chains from physical filler obstruction, as particulates impede solvent diffusion through the polymeric network, resulting in overestimated network connectivity measurements.

### 3.9. Evaluation of Crosslink Density—Mooney–Rivlin

**Figure 6:**
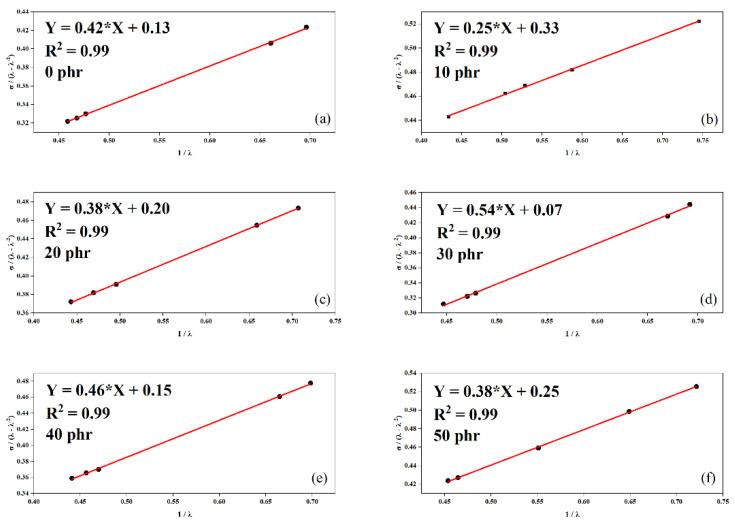
Plot of *σ*/(λ − λ^−2^) versus λ^−1^ for SBR/OLW composites with (**a**) 0 phr, (**b**) 10 phr, (**c**) 20 phr, (**d**) 30 phr, (**e**) 40 phr, and (**f**) 50 phr.

The crosslink density was established using the Mooney–Rivlin method, utilizing data derived from the tensile strength tests of the vulcanized SBR/OLW composites. Figure 6 presents a linear regression analysis for determining the material coefficients C_1_ and C_2_ following the Mooney–Rivlin theory.

The data presented in Table 6 indicate the crosslink densities, highlighting the increase in η values in accordance with the progressive incorporation of fillers into the composites. As established in Rooj et al.’s study [26], parameters C_1_ and C_2_ relate to the molecular network architecture and chain segment mobility correspondingly. The enhanced gradient in Mooney–Rivlin plots results from restricted chain deformability caused by polymer–filler interfacial effects, as reported in the literature [27,28]. The derived elastic constants, obtained from mechanical deformation analysis presented in Table 6, validate interfacial bonding between recycled lens material and the rubber matrix.

### 3.10. Filler–Matrix Compatibility Assessment via Lorenz–Parks Analysis

**Figure 7:**
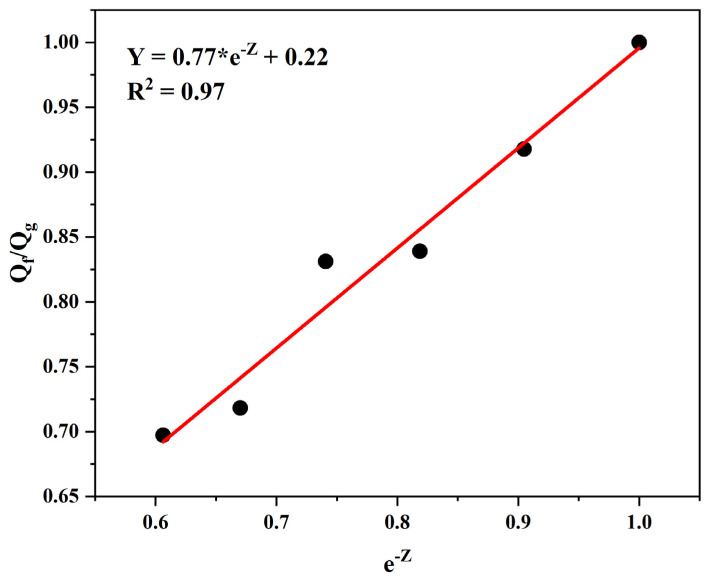
Dependence of Q_f_/Q_g_ ratio on exponential filler content for styrene–butadiene/ophthalmic waste blends with unfilled rubber as control.

The interfacial bonding characteristics between particulate fillers and the polymer network were quantified employing the Lorenz–Parks theoretical framework. In the graphical representation presented in Figure 7, the curves of Q_f_/Q_g_ vs. e^−z^ variation for the SBR composites containing OLW were plotted, with pure gum (without filler) as the reference.

The coefficients a and b, representing the empirical constants in the equation, demonstrate numerical solutions of 0.77 and 0.22, respectively, with a correlation factor R^2^ = 0.97. Following Lorenz–Parks’ theoretical framework, coefficient values exceeding 0.7 indicate significant interfacial adhesion between ophthalmic waste particles and the elastomeric network. Comparable findings for parameters a and b were reported by Santos et al. [21] in natural rubber-based composites containing collagenous residues.

**Figure 8:**
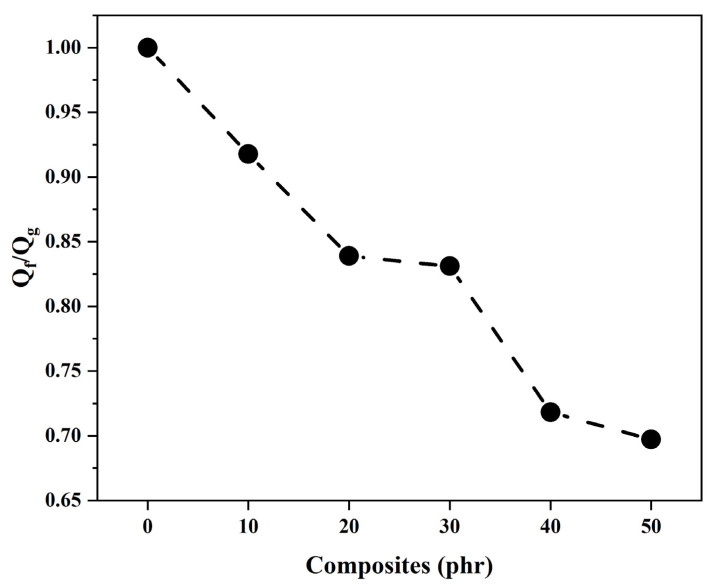
Effect of filler loading on Q_f_/Q_g_ of SBR/OLW composites and gum as reference.

Figure 8 reveals a progressive reduction in Q_f_/Q_g_ ratios with increasing OLW content, confirming strong filler–matrix compatibility.

### 3.11. Infrared Spectroscopy Assessment Using Attenuated Total Reflection

**Figure 9:**
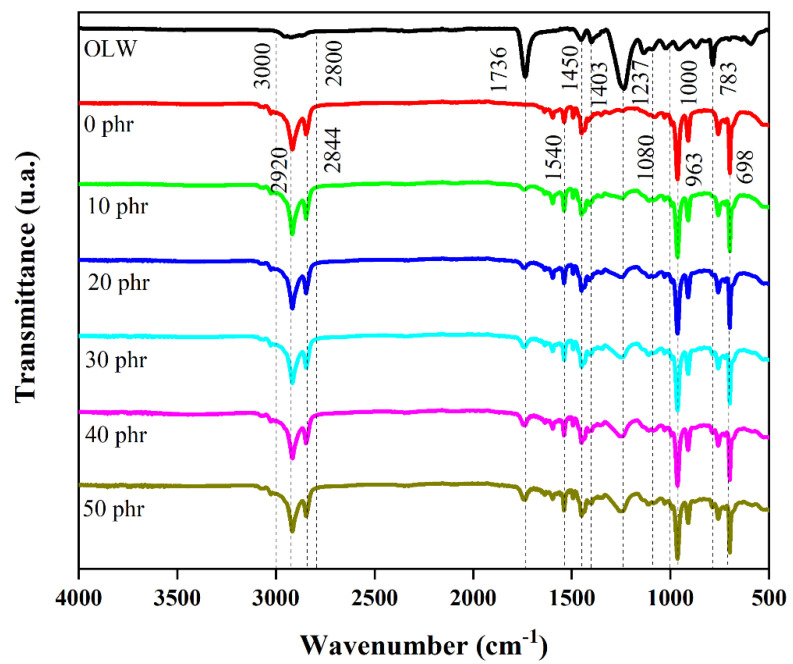
FTIR spectra of ophthalmic lens waste and SBR/OLW composites.

The infrared absorption profiles acquired for both ophthalmic lens residues and styrene–butadiene rubber formulations appear in Figure 9, with corresponding vibrational frequencies detailed in Table 7. Analysis of the FTIR spectrum of the OLW suggests a close resemblance to the spectrum of polymethyl methacrylate (PMMA) [29], albeit with the presence of some impurities, such as silica, originating from the grinding process of ophthalmic lenses, which are manufactured using silicon carbide or silicon dioxide abrasives. After vulcanization, no new spectra indicative of chemical reactions between the filler and the matrix are observed. Therefore, it can be inferred that the filler is adhered to or encapsulated by the matrix.

### 3.12. Thermal Decomposition Characterization

**Figure 10:**
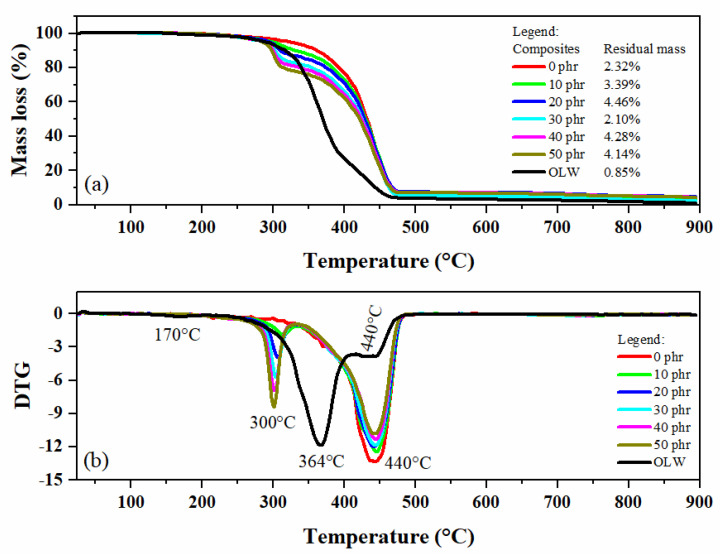
Curves of (**a**) TG of ophthalmic lens waste and SBR/OLW composites and (**b**) DTG of ophthalmic lens waste and SBR/OLW composites.

The heat resistance properties of the composite materials were examined using thermogravimetric evaluation. In Figure 10a, the mass loss curve of OLW is presented (represented by the black line). The material exhibits a three-phase decomposition process. Initial degradation commences near 170 °C, showing a weight reduction of 0.85% associated with chain scission of labile hydrogen bonds in the polymethyl methacrylate structure. The predominant decomposition phase appears at 364 °C, accounting for 77.62% mass loss linked to vinyl group depolymerization [35]. The final degradation stage emerges at 440 °C with 20.68% mass loss, representing oligomeric fragment decomposition [36]. The remaining char content from ophthalmic waste reaches about 0.85%, resulting from mineral components within the specimen, including silicon dioxide, bismuth oxide, tin compounds, brominated additives, etc., as detected through elemental fluorescence spectroscopy. In Figure 10a, the thermogram of the unfilled composite (represented by the red line) reveals a single, more intense degradation event occurring at 440 °C. This event corresponds to the degradation of organic compounds present in SBR rubber, leaving a residue of 2.32% of inorganic material [37]. It is noticeable that as more filler is added, there is a shift in the degradation temperatures of the composites to lower values.

Figure 10b shows the first derivative thermogravimetric (DTG) curves, where it is possible to clearly identify the degradation temperatures of each composite. In the filled composites (10–50 phr), two degradation events can be observed related to degradation temperatures: the first event occurs around 300 °C, corresponding to the degradation of the vinyl group in OLW, and the second event around 440 °C, attributed to the degradation of OLW oligomers and organic matter in SBR rubber.

### 3.13. Dynamic Mechanical Analysis (DMA)

**Figure 11:**
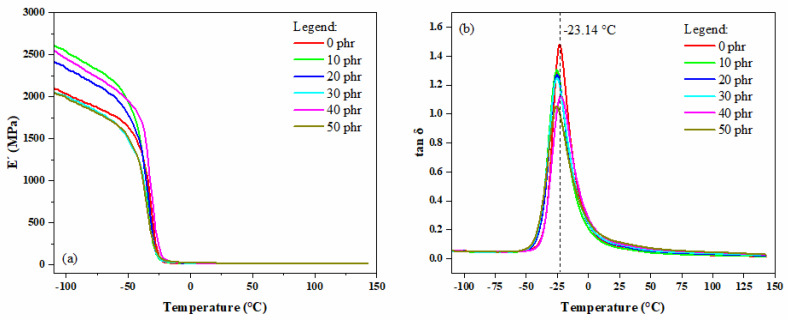
(**a**) Storage modulus (E’) curves and (**b**) tangent delta (tan δ) curves of the SBR/OLW composites.

The thermal behavior of storage modulus (E’) across different filler concentrations (0 to 50 phr) is illustrated in Figure 11a. At low temperatures, all the composites exhibit high E’ values, indicating significant material rigidity typical of the glassy state. As the temperature increases, there is a sharp decline in E’, representing the material’s transition to a more flexible and rubbery state (glass transition). With increasing OLW content, the initial E’ values also increase, suggesting that the addition of lens waste contributes to the greater rigidity of the composite. This rigidity results from the dispersion of OLW particles within the SBR matrix, which restricts the mobility of polymer chains. However, this restriction also reduces the material’s ability to dissipate energy under dynamic deformation, as evidenced by the decrease in the height of the tan δ peak. In applications where damping is essential (e.g., vibration isolation), this reduction may pose a limitation. After the glass transition, the composites reach a relatively low and constant E’ plateau characteristic of the rubbery state.

Figure 11b illustrates the temperature dependence of the damping coefficient (tan δ) for identical ophthalmic waste loadings. The maximum in the damping spectrum corresponds to the polymer’s glass–rubber transition temperature (Tg). As observed, the Tg of the composites occurs in the region around −23.14 °C, with very small variations among the different formulations. The height of the tan δ peak is related to the material’s damping capacity. A higher peak (as in the case of the composite without OLW, 0 phr) indicates greater energy dissipation, i.e., better damping capacity. As the OLW concentration increases, there is a slight decrease in the height of the tan δ peak, suggesting that the addition of OLW tends to reduce the composite’s damping capacity. However, the decrease in tan δ indicates that the interaction between the matrix and the filler may not be optimal, leading to lower efficiency in stress transfer and energy dissipation. This may be attributed to a potential lack of compatibility between OLW and SBR rubber or the formation of non-homogeneous agglomerates at higher OLW concentrations. The shift in the tan δ peak is minimal, indicating that the addition of lens waste does not significantly alter the glass transition temperature (Tg) but primarily affects the magnitude of the loss factor.

The practical implications of OLW usage vary depending on the application. In cases where damping is required, such as footwear soles and automotive components, the addition of OLW above 10 phr may compromise performance, with the OLW-free formulation (0 phr) offering the best balance between stiffness and energy dissipation. Conversely, in applications that prioritize stiffness, such as industrial coatings, higher OLW contents (30–50 phr) may be advantageous, as they enhance resistance to deformation without significantly altering Tg. To mitigate potential negative effects, several strategies can be adopted. Chemical modification of OLW, such as surface treatments via silanization, may improve adhesion between the matrix and the filler, preserving part of the damping capacity. The use of plasticizing agents above 2 phr can increase the mobility of polymer chains near the interface, compensating for the reduction in tan δ. Additionally, optimizing the concentration by limiting OLW content to ≤20 phr may provide a satisfactory balance between stiffness and damping, depending on the application’s requirements.

## 4. Comparative Study of Fillers in Vulcanized SBR Rubber Composites

Styrene–butadiene rubber (SBR) is a widely used synthetic elastomer in the industry due to its excellent processability, abrasion resistance, and low production cost. It is commonly employed in the manufacture of tires, footwear, automotive components, and coatings [38]. To enhance its performance, the incorporation of particulate materials—referred to as fillers—is a common strategy. These fillers may function as reinforcing or non-reinforcing (extender) agents, depending on their nature and the degree of interaction with the polymer matrix. Reinforcing fillers, such as carbon black and silica, significantly improve the mechanical and dynamic properties of the composite through efficient dispersion and strong interfacial adhesion with the rubber matrix. In contrast, non-reinforcing fillers, such as talc and calcium carbonate, are primarily added to reduce formulation costs and increase volume, offering limited or no structural reinforcement. The appropriate selection of filler type and its compatibility with SBR are essential in determining the final performance of the material, particularly in applications that demand high strength, flexibility, or damping capacity [39]. The following section presents scientific studies from the literature that explore the incorporation of fillers into the SBR rubber matrix, emphasizing their influence on the resulting properties of the composites.

Yang et al. [40] employed waste derived from synthetic running tracks (WSTP) as a reinforcing agent in styrene–butadiene rubber (SBR) composites. The researchers analyzed the composition of the waste and found it to be a mixture containing, by mass fraction, approximately 10.24% ethylene–propylene–diene rubber (EPDM), 6.47% paraffinic oil, and 75.77% calcium carbonate (CaCO_3_). In the composite formulations, 1.2 phr of bis(1-(tert-butylperoxy)-1-methylethyl)-benzene (BIBP) was incorporated—a bifunctional coupling agent capable of promoting chemical bonding between the organic phase of the SBR matrix and the inorganic phase of the waste material. This approach allowed for the incorporation of up to 90 phr of WSTP into the polymer matrix, resulting in significant improvements in mechanical properties. The unfilled composite exhibited a tensile strength of only 0.78 MPa and an elongation at break of 180%, attributed to the low molecular weight and reduced entanglement of the polymer chains. In contrast, the formulation containing 90 phr of WSTP demonstrated a tensile strength of 1.35 MPa and an impressive elongation at break of 1270%, which was attributed to the interaction between the rubber and the waste, indicating the formation of a type of physical crosslinking. This newly formed network contributed to better energy dissipation and, consequently, enhanced mechanical performance. The interfacial adhesion between the waste and the matrix was confirmed by scanning electron microscopy (SEM) images, which revealed a homogeneous distribution of WSTP particles within the SBR matrix. The authors emphasized that good filler dispersion favors improved mechanical performance. Furthermore, numerous particles were observed on the fracture surface of the composites; although some protruded from the surface, their contours with the matrix appeared blurred, indicating good compatibility. Larger particles were also embedded in the matrix, further suggesting satisfactory interfacial adhesion. This compatibility was attributed to the hydrophobic nature of WSTP, which favors interaction with the similarly nonpolar SBR matrix. Since the main component of WSTP is calcium carbonate, an inorganic material with a high modulus, this waste acted as a self-reinforcing filler in the composites. Consequently, increasing the WSTP content led to a gradual enhancement in the tensile strength of the composites. However, the strong interfacial adhesion also restricted the mobility of the SBR chains, which may result in decreased elongation at break with higher filler content. Swelling experiments were conducted by immersing the SBR/WSTP composites in toluene at room temperature for 24 h. It was observed that the degree of swelling increased with the WSTP content. Given that WSTP does not swell in toluene, this increase was attributed exclusively to the SBR matrix, indicating a reduction in its chemical crosslinking density. Thus, while mechanical strength benefited from the high modulus of the filler and the strong interfacial adhesion, the increased molecular mobility contributed to the enhanced elongation at break observed in the composites.

Abd El Aziz et al. [41] investigated the reinforcing effect provided by carbon black derived from agricultural waste biochar, used as a partial substitute for commercial carbon black in SBR rubber composites. As expected, the results were significant, given the inherently reinforcing nature of carbon black. The composite containing 100% commercial carbon black exhibited a tensile strength of 14.9 MPa and an elongation at break of 633%. In contrast, the composite formulated with 100% biochar-derived carbon black showed a tensile strength of 7 MPa and an elongation at break of 750%. Finally, the composite produced with an equal (50%) mixture of commercial and biochar-derived carbon black achieved a tensile strength of 11.2 MPa and an elongation at break of 700%. Scanning electron microscopy (SEM) analysis revealed that the progressive substitution of commercial carbon black with biochar promoted the formation of aggregates within the matrix. Crosslink density analyses, determined by swelling experiments in toluene, indicated that increasing the proportion of biochar led to a reduction in the rubber’s crosslink density. This behavior was attributed to the constant amount of curing agents used across all formulations.

Lopes et al. [42] evaluated the use of EVA (ethylene-vinyl acetate) waste incorporated into styrene–butadiene rubber (SBR) composites, aiming to propose a sustainable application for this residue within the footwear industry. The formulations were prepared with 10 and 20 parts per hundred rubbers (phr) of EVA waste, both at laboratory and industrial scales, and subjected to various tests to characterize their physical, mechanical, and rheological properties. Rheometric analyses indicated that the addition of EVA slightly affected the vulcanization parameters of the SBR matrix. A small increase in the scorch time (t_s_) was observed compared to the virgin material, along with a reduction in maximum torque (M_H_), suggesting a slight decrease in the crosslink density. Despite these variations, the changes were not deemed significant enough to require adjustments to the original formulation. In the laboratory-scale samples, the incorporation of EVA waste did not compromise properties such as flexural performance, density, or hardness, all remaining within the limits established by the Portuguese Footwear Technology Centre (CTCP). The density values ranged from 1.13 to 1.17 g cm^−3^, while hardness values varied between 57 and 62 Shore A—both suitable for outsole applications. Regarding abrasion behavior, an increase in material loss was recorded with higher EVA content, a result attributed to the presence of larger, less-integrated particles that detached more easily from the matrix. Nevertheless, all values remained below the CTCP’s upper limit of 250 mm^3^. With respect to tear resistance, SBR composites containing 10 phr of EVA exhibited values at the lower limit permitted by the standard, while those with 20 phr showed a slight decrease, particularly after thermal aging. Tensile strength followed a similar trend: a slight increase was observed at 10 phr, whereas a mild reduction occurred at 20 phr. Still, all measurements exceeded the required threshold of 8 MPa, indicating that the mechanical performance remained acceptable despite partial substitution by waste material. In industrial trials aimed at outsole production, the results were consistent with those obtained in the laboratory. Hardness and density remained stable, and flexural resistance was unaffected. Interestingly, the tear resistance of the outsoles incorporating EVA waste improved compared to laboratory-prepared sheets. This difference was attributed to the anisotropy induced by the multidirectional flow of material during molding, which may have promoted greater polymer chain entanglement. Tensile strength, although adequate, approached the minimum acceptable threshold, particularly in the composite containing 20 phr of EVA. Overall, the study demonstrated that incorporating up to 20 phr of EVA waste into SBR composites is technically feasible and compatible with the mechanical and physical requirements of the footwear industry. The waste acted as a non-reinforcing filler, providing moderate reinforcement in certain properties while contributing to slight reductions in others, such as tensile and tear strength. Nevertheless, the composites met all technical specifications, confirming the viability of sustainably reusing thermoset residues like EVA in higher value-added products.

Shanmugharaj et al. [43] compared the effects of pure silica (SiO_2_) and a functionalized hybrid silica–graphite (SiG) filler on the rheological, mechanical, and abrasion resistance performance of vulcanized SBR composites. The results demonstrated that the silica–graphite filler, synthesized via chemical grafting of silica onto expanded graphite, provided significantly superior reinforcement compared to pure silica. Specifically, the incorporation of 20 parts per hundred rubber (phr) of SiG led to an approximate 160% increase in the 100% modulus relative to unfilled SBR, whereas the same loading of pure silica resulted in only a 50% increase. This substantial difference indicates a considerable enhancement in material stiffness when using the hybrid filler, which is attributed to the stronger interaction between the polymer matrix and the filler, as well as to the lamellar morphology of graphite that promotes mechanical interlocking and more effective stress transfer. Tensile strength was also positively influenced. With 20 phr of pure silica, a 33% increase in tensile strength was observed, while the same amount of SiG produced a 45.5% improvement. Although elongation at break showed a decreasing trend with higher filler content—an expected outcome due to the presence of rigid domains that facilitate crack propagation—all values remained suitable for elastomeric applications. Complementary bound rubber tests revealed a higher amount of physically entrapped rubber at the filler–elastomer interface in composites containing SiG compared to those with pure silica. The bound rubber content increased by approximately 20% in the SiG systems, indicating a more cohesive and effective interface for mechanical load transfer. Additionally, composites filled with SiG exhibited lower swelling indices in toluene, suggesting a denser network structure consistent with a higher degree of physical and/or chemical entanglement between the filler and the matrix. These findings were corroborated by scanning electron microscopy (SEM) micrographs, which revealed better dispersion of SiG particles within the SBR matrix, with average sizes below 5 μm, whereas pure silica formed agglomerates up to 10 μm in size. Furthermore, abrasion performance was markedly improved in the SiG-containing composites. For the formulation with 40 phr, abrasion loss was reduced by 59% compared to neat SBR and was significantly lower than the 32% reduction observed with conventional silica. Overall, the results indicate that silica–graphite acts as a highly efficient reinforcing filler for SBR, delivering substantial enhancements in mechanical properties and wear resistance. The synergistic effect between the lamellar structure of graphite and the functional groups of silica not only promotes better interfacial coupling but also introduces a geometric reinforcement effect not observed with traditional fillers such as pure silica or carbon black. These findings are promising for the development of high-performance and more sustainable elastomeric composites, with potential applications in tires, footwear, and other sectors requiring elevated mechanical strength combined with enhanced durability.

In our study, 2 phr of the coupling agent Chartwell^®^ was employed. The results demonstrated that the incorporation of ophthalmic lens waste (OLW) into styrene–butadiene rubber (SBR) composites led to significant improvements in several physico-mechanical and thermal properties, particularly in formulations containing between 10 and 20 phr of OLW. These compositions proved to be the most balanced in terms of mechanical performance and stability and were identified as the most suitable for practical applications such as the production of footwear soles and rubberized flooring. Regarding mechanical properties, the addition of 10 phr OLW resulted in the highest elongation at break (236.74 ± 27.76%), surpassing even the control composite (without OLW), which exhibited 184.02 ± 23.78%. Moreover, this formulation also showed the highest tensile strength among the evaluated composites (1.34 ± 0.16 MPa), indicating that, at low concentrations, OLW can positively contribute to the mechanical strength of the material. Shore A hardness analysis revealed a progressive increase in the OLW content, indicating a rise in the surface stiffness of the composites. This behavior was accompanied by an increase in density, which rose from approximately 1.0 to 1.1 g·cm^−3^ with the incorporation of up to 50 phr OLW. These characteristics are desirable in applications that demand higher resistance to surface wear, such as footwear and industrial coatings. Morphological analysis via scanning electron microscopy (SEM) revealed good dispersion of OLW particles within the SBR matrix up to 30 phr, with homogeneous distribution and a well-defined interface. Above this concentration, agglomerations and the formation of stress concentration points were observed, which may compromise mechanical performance. With respect to crosslink density, both the swelling method (Flory–Rehner) and mechanical analysis (Mooney–Rivlin) indicated a systematic increase in the polymer network connectivity upon OLW addition. The crosslink density ranged from 1.31 × 10^−4^ mol·cm^−3^ (0 phr) to 1.47 × 10^−4^ mol·cm^−3^ (50 phr), suggesting that OLW acts as a restricting agent on polymer chain mobility. However, FTIR spectroscopic analysis did not detect the formation of chemical bonds between OLW and the polymer matrix, indicating that the interaction occurs predominantly through physical effects and surface adhesion. Thermal analysis by thermogravimetry (TG) showed that the incorporation of OLW did not compromise the thermal stability of the composites. The samples exhibited two degradation stages associated with the decomposition of OLW and the SBR matrix, maintaining degradation temperatures above 300 °C—an acceptable behavior for various industrial applications. Finally, dynamic mechanical analysis (DMA) revealed that OLW-containing composites exhibited an increase in storage modulus (E’), confirming greater stiffness. However, there was a reduction in damping capacity, evidenced by the decrease in the tan δ peak. Despite this, the glass transition temperature (Tg) remained virtually unchanged (~−23.14 °C), indicating that the addition of OLW did not significantly affect the thermal performance of the material. Therefore, the formulation containing 10 to 20 phr of OLW represents the best combination of properties, being both technically feasible and environmentally advantageous for the production of vulcanized SBR composites intended for applications such as soles, coatings, and general-purpose materials.

## 5. Conclusions

The research in question investigated the feasibility of incorporating OLW into polymeric composites, aiming to develop more sustainable and economically viable solutions. One of the main aspects analyzed was the impact of OLW addition on the vulcanization parameters of SBR. The findings revealed that the OLW additive minimally affected the curing kinetics, offering potential energy efficiency benefits during production. Moreover, the waste material incorporation elevated the network density within the elastomeric matrix, as quantified through both swelling equilibrium and stress–strain analysis. These observations imply interfacial bonding between the particulate filler and styrene–butadiene elastomer, though insufficient to substantially improve the material’s load-bearing capacity, primarily functioning as a space-filling component. The analysis of matrix–filler interaction using the Lorenz–Parks model supported the observation of interaction between OLW and SBR rubber. However, such molecular interactions did not translate into meaningful improvement of the composite’s structural performance.

In the thermogravimetric tests, the composites containing OLW demonstrated good thermal stability, which is a desirable characteristic for various applications. In contrast, dynamic mechanical analysis shows that the addition of OLW to the SBR composite increases the material’s rigidity, as evidenced by the rise in the storage modulus (E’). However, this addition slightly reduces the composite’s damping capacity, as indicated by the decrease in the tan δ peak. The polymer’s viscoelastic transition point shows minimal variation, demonstrating that the heat-related characteristics of the formulated material are largely unaltered by the incorporated optical waste, while the structural and energy dissipation capabilities are modified.

Additionally, infrared spectroscopy did not detect significant chemical interactions between the polymer matrix and the filler. Based on the results obtained, it was possible to identify that the optimal compositions of OLW are in the range of 10 to 20 phr. This information enabled the production of a new vulcanized composite, demonstrating the feasibility of its application.

In summary, incorporating ophthalmic lens residues as reinforcing particulates in crosslinked styrene–butadiene elastomer formulations offers a viable approach toward developing eco-friendly and cost-effective material solutions. This study contributes to advancing knowledge on the reuse of industrial waste in the polymer industry, promoting more efficient and environmentally responsible practices.

## Figures and Tables

**Table 1:** Formulation of SBR polymer composites with recycled ophthalmic lens waste.

Formulation Components	Quantities of Components in phr *					
	SBR/OLW_0_	SBR/OLW_10_	SBR/OLW_20_	SBR/OLW_30_	SBR/OLW_40_	SBR/OLW_50_
SBR 1502	100	100	100	100	100	100
Zinc oxide	5	5	5	5	5	5
Stearic acid	3	3	3	3	3	3
PEG 4000	2	2	2	2	2	2
Chartwell^®^	2	2	2	2	2	2
Naphthenic oil	10	10	10	10	10	10
OLW filler	0	10	20	30	40	50
Sulfur	1.5	1.5	1.5	1.5	1.5	1.5
MBTS	1.2	1.2	1.2	1.2	1.2	1.2
TMTD	0.3	0.3	0.3	0.3	0.3	0.3
Total	125	135	145	155	165	175

* phr (per hundred rubber); SBR/OLW (SBR rubber composite and ophthalmic lens waste).

**Table 2:** Rheological characteristics of styrene–butadiene/OLW composites.

SBR/OLW Composites	M_L_ (dNm)	M_H_ (dNm)	ΔM = (M_H_ − M_L_)(dNm)	t_s_ (min)	t_90_ (min)
0 phr	5.34 ± 0.11	27.62 ± 1.94	22.29 ± 1.87	1.94 ± 0.03	3.83 ± 0.16
10 phr	5.71 ± 0.07	28.36 ± 0.76	22.65 ± 0.81	1.85 ± 0.01	3.88 ± 0.39
20 phr	6.27 ± 0.07	29.40 ± 0.96	23.13 ± 1.00	1.82 ± 0.01	3.80 ± 0.35
30 phr	6.51 ± 0.08	29.69 ± 0.96	23.18 ± 0.99	1.79 ± 0.02	3.74 ± 0.22
40 phr	6.89 ± 0.00	29.63 ± 0.59	22.74± 0.59	1.93 ± 0.03	4.26 ± 0.33
50 phr	7.26 ± 0.11	31.24 ± 0.85	23.98 ± 0.91	1.84 ± 0.03	3.88 ± 0.40

**Table 3:** Molecular constituents in ophthalmic waste materials analyzed by X-ray fluorescence.

Ophthalmic Lens Waste	
Chemical Element	Quantity (%)
C	98.128
S	0.776
Si	0.551
Bi	0.180
In	0.110
Sn	0.052
Br	0.030
Ca	0.030
K	0.013
Fe	0.012
Cu	0.008
Zn	0.003
Pb	0.011
Others	0.096
Total	100.000

**Table 4:** Fracture resistance test results.

Composites	M 100% (MPa)	M 200% (MPa)	Tensile at Break (MPa)	Strain at Break (%)
SBR/OLW_0_	0.8	---	1.20 ± 0.15	184.02 ± 23.78
SBR/OLW_10_	0.76	1.2	1.34 ± 0.16	236.74 ± 27.76
SBR/OLW_20_	0.68	1.06	1.13 ± 0.13	206.72 ± 25.41
SBR/OLW_30_	0.66	1.04	1.08 ± 0.14	208.74 ± 18.61
SBR/OLW_40_	0.58	0.93	1.12 ± 0.13	232.71 ± 37.31
SBR/OLW_50_	0.57	0.88	0.91 ± 0.15	208.59 ± 37.45

**Table 5:** Network density determination via solvent swelling (Flory–Rehner approach).

Composites	Flory–Rehner
	ν × 10^−4^ (mol cm^−3^)
SBR/OLW_0_	1.31
SBR/OLW_10_	1.37
SBR/OLW_20_	1.40
SBR/OLW_30_	1.40
SBR/OLW_40_	1.43
SBR/OLW_50_	1.47

**Table 6:** Network connectivity assessment via Mooney–Rivlin analysis.

Composites	Mooney–Rivlin		
	η × 10^−4^ (mol cm^−3^)	C_1_	C_2_
SBR/OLW_0_	4.44	0.26	0.85
SBR/OLW_10_	4.73	0.67	0.51
SBR/OLW_20_	4.73	0.40	0.77
SBR/OLW_30_	4.91	0.14	1.08
SBR/OLW_40_	4.97	0.31	0.92
SBR/OLW_50_	5.1	0.50	0.77

**Table 7:** FTIR spectra bands of ophthalmic lens waste and SBR/OLW composites.

Ophthalmic Lens Waste (OLW)		
Wavenumber (cm^−1^)	Assignments	Reference
3000–2800	Symmetric and asymmetric C-H stretching of PMMA	[30]
1736	C=O stretching vibration of PMMA	[30]
1450	CH_3_ bending of PMMA	[30]
1403	C-H stretching of PMMA	[30]
1237	C-O-C stretching of PMMA	[30]
1130	Symmetric C-H stretching of PMMA	[31]
1080	Si-O stretching (impurity—sanding process)	[32]
783	C-H bending of PMMA	[30]
**Styrene–Butadiene Rubber (SBR)**		
**Wavenumber (cm^−1^)**	**Assignments**	**Reference**
2920	Aromatic ring C-H stretching	[27]
2844	Aromatic ring C-H stretching	[27]
1540	CH_2_ bending	[27]
1450	CH_3_ bending	[27]
1080	Si-O stretching (impurity and added coupling agent)	[32]
963	Aromatic ring C-C stretching	[33]
698	Aromatic ring C-C stretching	[34]

## Data Availability

The original contributions presented in this study are included in the article. Further inquiries can be directed to the corresponding author.

## References

[1] World Health Organization (WHO) (2019). World Report on Vision. https://cdn.who.int/media/docs/default-source/documents/publications/world-vision-report-accessible.pdf?sfvrsn=223f9bf7_2.

[2] Zion Market Research (2022). Single Vision Lenses Market. https://www.zionmarketresearch.com/report/single-vision-lenses-market.

[3] Tech Navio Ophthalmic Lens Market by End-User, Product and Geography—Forecast and Analysis 2023–2027. https://www.technavio.com/report/ophthalmic-lens-market-industry-analysis.

[4] Schirmeister C.G., Mülhaupt R. (2022). Closing the carbon loop in the circular plastics economy. Macromol. Rapid Commun..

[5] Pillay R., Hansraj R., Rampersad N. (2023). Spectacle lens and contact lens recycling in South Africa. Afr. Vis. Eye Health.

[6] Encarnação T., Nicolau N., Ramos P., Silvestre E., Mateus A., de Carvalho T.A., Gaspar F., Massano A., Biscaia S., Castro R.A.E. (2023). Recycling Ophthalmic Lens Wastewater in a Circular Economy Context: A Case Study with Microalgae Integration. Materials.

[7] Batista S.S., de Souza L.G.M., de Lima Bezerra D.M., Neto R.V.P. (2020). Viabilities for obtaining, manufacturing and applying composites using bamboo powders and ophthalmic lens waste. Res. Soc. Dev..

[8] (2021). Standard Practice for Rubber—Materials, Equipment, and Procedures for Mixing Standard Compounds and Preparing Standard Vulcanized Sheets.

[9] (2019). Standard Test Method for Rubber Property—Vulcanization Using Oscillating Disk Cure Meter.

[10] Lee B.L. (1979). Reinforcement of uncured and cured rubber composites and its relationship to dispersive mixing—An interpretation of cure meter rheographs of carbon black loaded SBR and cis-polybutadiene compounds. Rubber Chem. Technol..

[11] (2022). Standard Test Methods for Rubber Products—Chemical Analysis.

[12] Flory P.J., Rehner J. (1943). Statistical mechanics of cross-linked polymer networks I. Rubberlike elasticity. J. Chem. Phys..

[13] Mooney M. (1940). A theory of large elastic deformation. J. Appl. Phys..

[14] Gruendken M., Koda D., Dryzek J., Blume A. (2021). Low molecular weight ‘liquid’ polymer extended compounds, impact on free volume and crosslink density studied by positron lifetime spectroscopy and stress-strain analysis according to Mooney-Rivlin. Polym. Test..

[15] Sombatsompop N. (1998). Practical use of the Mooney-Rivlin equation for determination of degree of crosslinking of swollen nr vulcanisates. J. Sci. Soc. Thail..

[16] (2021). Standard Test Methods for Vulcanized Rubber and Thermoplastic Elastomers—Tension.

[17] (2020). Standard Test Method for Tear Strength of Conventional Vulcanized Rubber and Thermoplastic Elastomers.

[18] (2021). Standard Test Method for Rubber Property—Durometer Hardness.

[19] (2019). Standard Test Method for Rubber Property—Abrasion Resistance (Rotary Drum Abrader).

[20] Lorenz O., Park C.R. (1961). The crosslinking efficiency of some vulcanizing agents in natural rubber. J. Polym. Sci..

[21] Santos R.J., Hiranobe C.T., Dognani G., Silva M.J., Paim L.L., Cabrera F.C., Torres G.B., Job A.E. (2022). Using the Lorenz–Park, Mooney–Rivlin, and dynamic mechanical analysis relationship on natural rubber/leather shavings composites. J. Appl. Polym. Sci..

[22] (2019). Standard Test Method for Rubber—Compositional Analysis by Thermogravimetry (TGA).

[23] Najam M., Hussain M., Ali Z., Maafa I.M., Akhter P., Majeed K., Ahmed A., Shehzad N. (2020). Influence of silica materials on synthesis of elastomer nanocomposites: A review. J. Elastomers Plast..

[24] Kim D.Y., Park J.W., Lee D.Y., Seo K.H. (2020). Correlation between the crosslink characteristics and mechanical properties of natural rubber compound via accelerators and reinforcement. Polymers.

[25] Hiranobe C.T., Ribeiro G.D., Torres G.B., dos Reis E.A.P., Cabrera F.C., Job A.E., Paim L.L., dos Santos R.J. (2021). Cross-linked density determination of natural rubber compounds by different analytical techniques. Mater. Res..

[26] Rooj S., Das A., Heinrich G. (2011). Tube-like natural halloysite/fluoroelastomer nanocomposites with simulta-neous enhanced mechanical, dynamic mechanical and thermal properties. Eur. Polym. J..

[27] Schieppati J., Schrittesser B., Wondracek A., Robin S., Holzner A., Pinter G. (2021). Temperature impact on the mechanical and fatigue behavior of a non-crystallizing rubber. Int. J. Fatigue.

[28] He S., Hu J., Zhang C., Wang J., Chen L., Bian X., Lin J., Du X. (2018). Performance improvement in nano-alumina filled silicone rubber composites by using vinyl tri-methoxysilane. Polym. Test..

[29] Al-Harbi F.A., Abdel-Halim M.S., Gad M.M., Fouda S.M., Baba N.Z., AlRumaih H.S., Akhtar S. (2019). Effect of nanodiamond addition on flexural strength, impact strength, and surface roughness of PMMA denture base. J. Prosthodont..

[30] Kaur J., Sharma J.P., Singh N., Pathak D., Guleria N., Singh P.K., Sharma P.K. (2023). Improvement in optical absorption and emission characteristics of polymethyl methacrylate in solution cast polymethyl methacrylate/polyvinyl carbazole polyblends. J. Thermoplast. Compos. Mater..

[31] Singh S., Arora N., Paul K., Kumar R., Kumar R. (2019). FTIR and rheological studies of PMMA-based nano-dispersed gel polymer electrolytes incorporated with LiBF_4_ and SiO_2_. Ionics.

[32] Hayeemasae N., Waesateh K., Masa A., Ismail H. (2019). Halloysite nanotubes filled natural rubber composites: Functionality, crystallinity and thermal studies. J. Eng. Sci..

[33] Baeta D.A., Zattera J.A., Oliveira M.G., Oliveira P.J. (2009). The use of styrene-butadiene rubber waste as a potential filler in nitrile rubber: Order of addition and size of waste particles. Braz. J. Chem. Eng..

[34] Lin T., Zhu L., Chen W., Wu S., Guo B., Jia D. (2013). Reactivity of sulfide-containing silane toward boehmite and in situ modified rubber/boehmite composites by the silane. Appl. Surf. Sci..

[35] Nikolaidis A.K., Achilias D.S. (2018). Thermal degradation kinetics and viscoelastic behavior of poly (methyl methacrylate)/organomodified montmorillonite nanocomposites prepared via in situ bulk radical polymerization. Polymers.

[36] Kashiwagi T., Inaba A., Brown J.E., Hatada K., Kitayama T., Masuda E. (1986). Effects of weak linkages on the thermal and oxidative degradation of poly (methyl methacrylates). Macromolecules.

[37] Ali M.A.M., Raslan H.A., El-Nemr K.F., Hassan M.M. (2020). Thermal and mechanical behavior of SBR/devulcanized waste tire rubber blends using mechano–chemical and microwave methods. J. Polym. Eng..

[38] Ansari A., Mohanty T.R., Sarkar S., Ramakrishnan S., Amarnath S., Singha N.K. (2024). Epoxy modified styrene butadiene rubber (SBR) in green tire application. Eur. Polym. J..

[39] Yadav R., Singh M., Shekhawat D., Lee S.-Y., Park S.-J. (2023). The role of fillers to enhance the mechanical, thermal, and wear characteristics of polymer composite materials: A review. Compos. Part A Appl. Sci. Manuf..

[40] Yang W., Li Y., Chen Y., Lu Y., Jiang X., Cui P., Hao W. (2023). Upcycling waste synthetic running tracks in reinforcement of styrene-butadiene rubber. J. Clean. Prod..

[41] Abd El-Aziz M.E., Shafik E.S., Tawfic M.L., Morsi S.M.M. (2022). Biochar from waste agriculture as reinforcement filer for styrene/butadiene rubber. Polym. Compos..

[42] Lopes D., Ferreira M.J., Russo R., Dias J.M. (2015). Natural and synthetic rubber/waste–Ethylene-Vinyl Acetate composites for sustainable application in the footwear industry. J. Clean. Prod..

[43] Shanmugharaj A.M., Kumar K.T., Sundari G.S., Kumar E.S., Ashwini A., Ramya M., Varsha P., Kalaivani R., Raghu S., Ryu S. (2019). Study on the effect of silica–graphite filler on the rheometric, mechanical, and abrasion loss properties of styrene–butadiene rubber vulcanizates. J. Elastomers Plast..

